# Inflammatory diseases of the aorta: Part 1: Non-infectious aortitis

**DOI:** 10.1007/s00772-016-0143-9

**Published:** 2016-06-08

**Authors:** I. Töpel, N. Zorger, M. Steinbauer

**Affiliations:** 1 Klinik für Gefäßchirurgie, Krankenhaus Barmherzige Brüder, Prüfeninger Str. 86, 93049 Regensburg, Germany; 2 Institut für Diagnostische und Interventionelle Radiologie und Neuroradiologie, Krankenhaus Barmherzige Brüder, Regensburg, Germany

**Keywords:** Pathogenesis, Vasculitis, Arteritis, Aortic infection, Rheumatic diseases, Pathogenese, Vaskulitis, Arteriitis, Infektion der Aorta, Rheumatische Erkrankungen

## Abstract

Aortitis is a term which encompasses inflammatory changes to the aortic wall from various pathogenic etiologies. Large vessel vasculitis, such as Takayasu arteritis and giant cell arteritis represent the most common entities; however, there is also an association with other rheumatological diseases. Chronic idiopathic periaortitis represents a distinct disease entity and infectious aortitis is a rare but life-threatening disease. Due to the diverse clinical pictures vascular surgeons often face a significant challenge in terms of making an accurate initial diagnosis. Treatment requires an interdisciplinary approach. This article describes the pathogenesis of the various forms of aortitis as well as the diagnostic methods and treatment approaches.

## Zusammenfassung

Unter dem Begriff der Aortitis werden entzündliche Veränderungen der Aortenwand verschiedener pathogenetischer Ursachen zusammengefasst. Großgefäßvaskulitiden, wie die Takayasu-Arteriitis und die Riesenzellarteriitis, stellen die häufigsten Erkrankungen dar, es findet sich aber auch eine Assoziation mit anderen rheumatologischen Krankheiten. Die chronische idiopathische Periaortitis stellt eine eigene Entität dar. Eine infektiöse Aortitis ist eine seltene aber lebensbedrohliche Situation. Für den Gefäßmediziner ist es auf Grund des vielgestaltigen klinischen Bildes oft schwierig, eine exakte Erstdiagnose zu stellen. Die Behandlung erfordert eine interdisziplinäre Zusammenarbeit. Dieser Beitrag soll Ursachen, diagnostische Methoden und Therapieansätze der verschiedenen Aortitiden darstellen.

## Study goal

Inflammatory changes to the aortic wall can be caused by a multitude of different diseases. The broad spectrum of pathogenic factors and nonspecific clinical presentation often delay initial diagnosis; however, a precise determination of the underlying pathology is essential for successful management. The aim of this article is to describe the pathogenesis, symptoms and diagnosis of aortitis and equip the reader with the knowledge required to perform adequate treatment following an appropriate diagnostic work-up.

### Definition and classification

The term aortitis encompasses inflammatory changes to the aortic wall from varying etiologies that are associated with certain specific histopathological lesions. The inflammatory response may be limited to the aortic wall or accompanied by changes directly adjacent to the vessel (e. g. retroperitoneal fibrosis) or other vessels (e. g. giant cell arteritis). A distinction is made between infectious and non-infectious aortitis. Takayasu arteritis and giant cell arteritis are the most common causes of non-infectious aortitis [6, 7]; however, an association has been shown between other rheumatological diseases and inflammatory changes to the aortic wall, e. g. rheumatoid arthritis, systemic lupus erythematosus, Wegener’s granulomatosis and Behçet’s disease (Tab. 1, [2]). It is essential to differentiate between non-infectious causes of aortitis and the much rarer infectious causes, as treatment approaches differ greatly. Numerous bacteria have been described as causative agents in infectious inflammation, the most common being *Salmonella, Staphylococci *and *Pneumococci *[4, 16]*.* More rarely, aortic wall infections may be caused by mycobacteria that infiltrate the aortic wall from infected lung tissue, lymph nodes or via milia [22]; however, fungi and viruses have also been described as causative agents in aortitis [8, 20]. Part 2 of this continuing medical education (CME) article provides a detailed description of infectious aortitis.

**Tab. 1:** Forms of underlying rheumatic diseases associated with inflammatory lesions of the aorta

Rheumatoid arthritis
Systemic lupus erythematosus
HLA B27-associated spondyloarthropathies
Antineutrophil cytoplasmic antibodies (ANCA)-associated vasculitis
Wegener’s disease
Panarteritis nodosa syn.
Behçet’s disease
Sarcoidosis
Cogan’s syndrome
Reiter’s syndrome

## Non-infectious aortitis

### Takayasu arteritis

Takayasu arteritis (synonyms: pulseless disease, occlusive thromboaortopathy and Martorell syndrome) is a chronic inflammatory arteritis primarily affecting the large vessels, in particular the aorta and its branches. Although case reports date back as far as 1830 it was not until 1905 that the characteristic fundal lesions were published by Takayasu, a professor of ophthalmology at the Kanazawa University in Japan as ischemic neuropathy of the optic nerve with annular arteriovenous neo-anastomosis. The disease is rare, with 5000 cases reported throughout Japan between 1990 and 2000, while a US study put the incidence at 2.6 cases per 1,000,000 inhabitants per year. The incidence in European countries is unknown. The majority of cases are still seen in East Asia where young females are almost exclusively affected [5]. A phase of asymptomatic disease generally precedes clinically apparent symptoms and findings. Disease onset is generally seen in the second or third decade of life, whereby the interval between symptom onset and diagnosis may be protracted (range 2–11 years [17]). Nonspecific symptoms include fever, nocturnal sweating, malaise, weight loss, joint and muscle pain as well as mild anemia. As vascular lesions progress, stenosis and occlusion occur with resultant ischemia to end organs [7]. Changes in the aortic region affect the abdominal section in particular and need to be differentiated from other causes of atypical coarctation of the aorta (e.g. mid-aortic syndrome) (Tab. 2). The aortic arch and its branches are affected almost equally. Characteristic features include weakened or absent peripheral pulse (pulseless disease), vascular bruits, renal hypertension due to renal artery stenosis, retinopathy, aortic valve insufficiency due to dilation of the valve ring and subsequent dilated cardiomyopathy and myocardial ischemia due to coronary ostial stenosis. Syncope, epileptic seizures and amaurosis fugax may occur as a result of ischemic or hypertensive cerebral damage. Carotidynia and erythema nodosum are rare clinical findings that may be linked to Takayasu arteritis.

**Tab. 2:** Differential diagnosis of mid-aortic syndrome

Congenital	Abdominal aortic coarctation
Acquired	Neurofibromatosis (Recklinghausen disease)
	Takayashu arteritis
	Giant cell arteritis

**Fig. 1:**
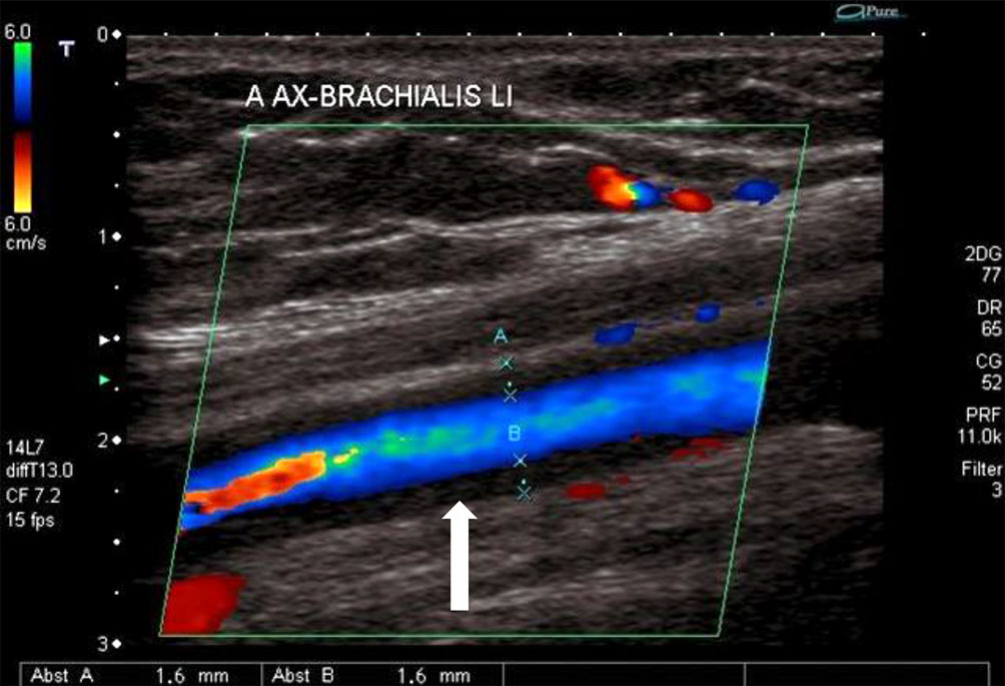
Takayasu arteritis with brachial artery involvement and typical halo (*arrow*) on color duplex sonography (courtesy of Dr. G. Herzog)

**Fig. 2:**
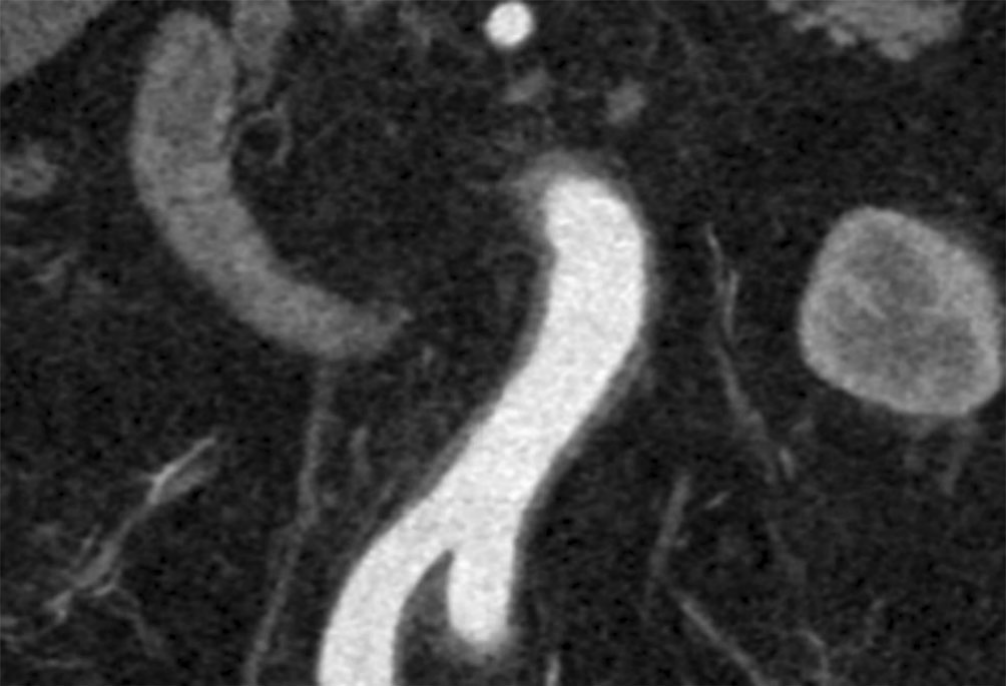
Widening of the infrarenal aortic wall in a female patient with Takayasu arteritis

**Fig. 3:**
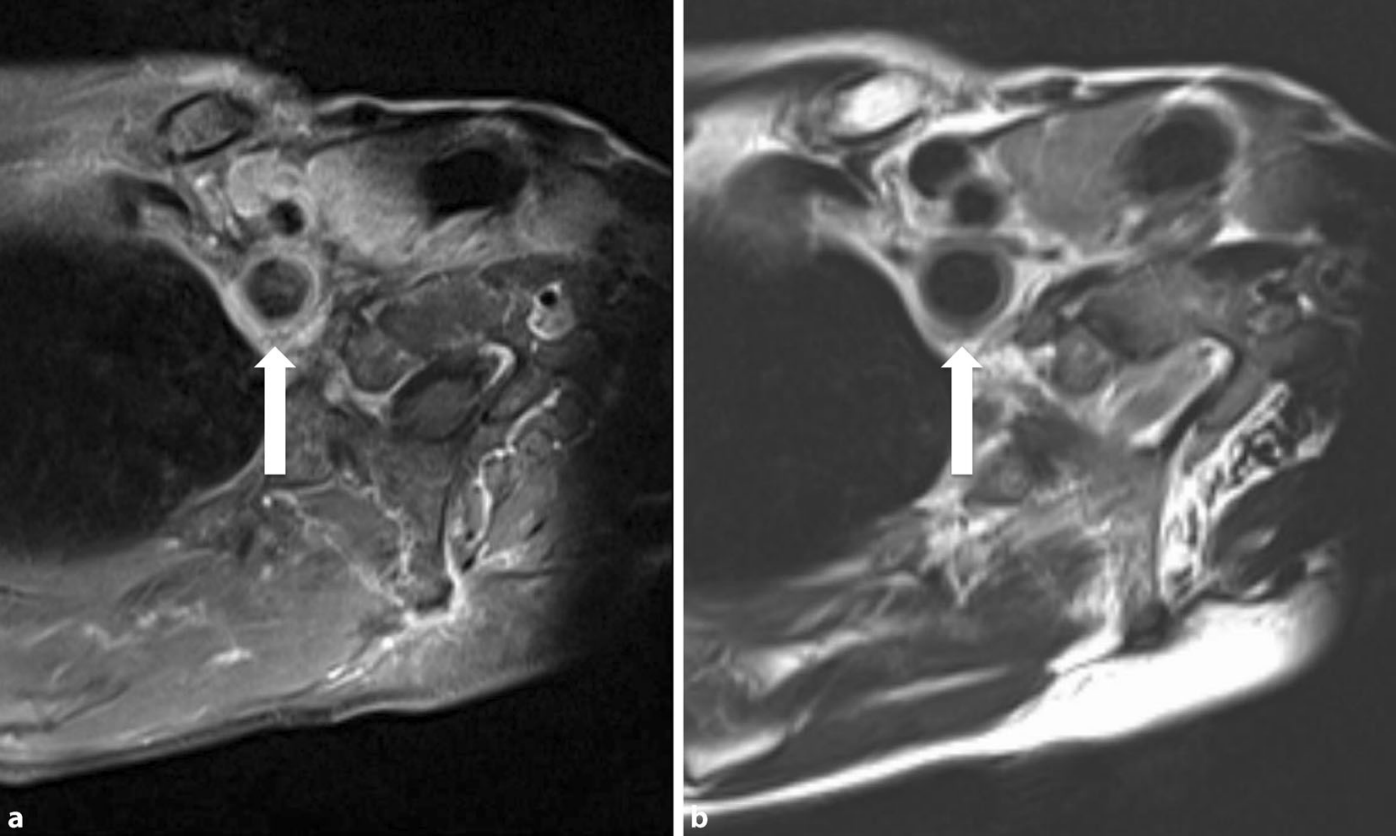
Magnetic resonance imaging showing **(a)** signal enhancement and **(b) **marked widening of the subclavian artery wall in a female patient with Takayasu arteritis (*arrows*)

**Fig. 4:**
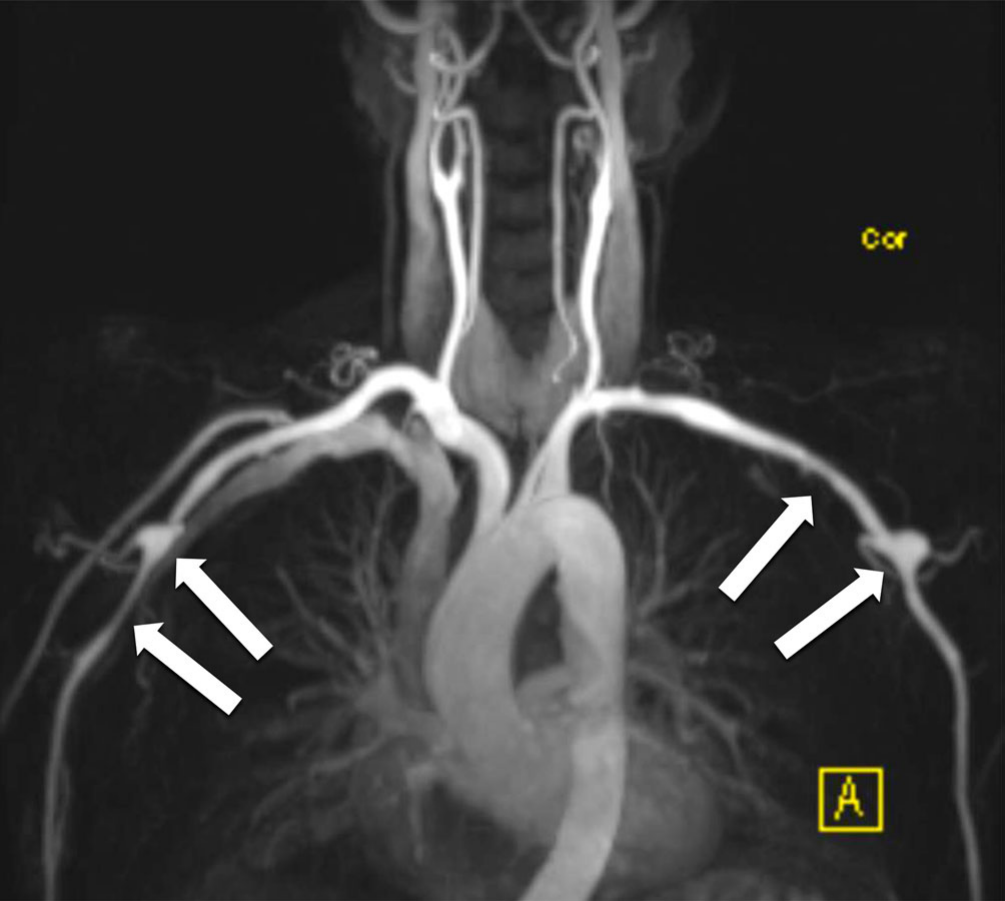
Magnetic resonance angiography in the same patient as in Fig. 3 showing inflammatory aneurysms of the axillary arteries (*arrows*)

**Fig. 5:**
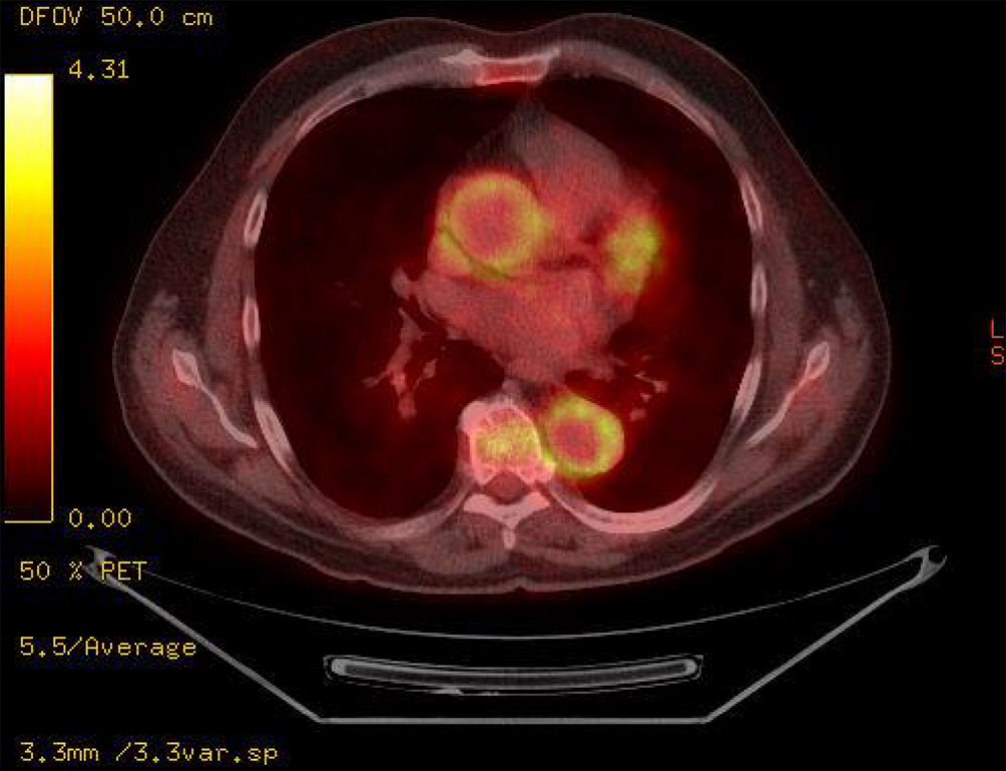
Female patient with Takayasu arteritis and positive positron emission tomography computed tomography showing marked enhancement around the aortic wall

In view of the frequently observed manifestation of decreased perfusion of internal organs or extremities, (duplex) ultrasound plays an important role in the basic diagnostic work-up and can provide valuable early information about the correct diagnosis by differentiating between stenotic morphology and arteriosclerotic lesions (Fig. 1); nevertheless, modern ultrasound methods are not yet able to replace cross-sectional diagnostic imaging. Tab. 3 lists the specific diagnostic criteria defined by the American College of Rheumatology (ACR). Computed tomography (CT) angiography enables visualization of the characteristic pattern of involvement of the aorta and its branches, while at the same time permitting an assessment of the extent of inflammatory changes to the vessel walls (Fig. 2). Digital subtraction angiography (DSA) should only be used in the case of specific questions or for interventional purposes. Although vascular imaging by means of contrast-enhanced magnetic resonance imaging (MRI) is possible, assessing the vessel wall and neighboring structures may be limited due to lower spatial resolution depending on the acquisition technique used ([28], Figs. 3 and 4). Positron emission tomography-computed tomography (PET-CT) plays an increasingly important role in the assessment of inflammatory activity ([14], Fig. 5).

**Tab. 3:** American College of Rheumatology criteria for the classification criteria of Takayasu arteritis which is highly probable in the presence of three or more criteria (sensitivity 91 % and specificity 98 % [33])

Age at disease onset < 40 years
Claudication of the extremities
Weak brachial artery pulse
Blood pressure difference > 10 mmHg between arms
Auscultable stenotic bruits over the subclavian artery or abdominal aorta
Pathognomonic angiographic findings

While it is possible to classify patients based on the pattern of aortic and aortic branch involvement, this does not contribute to assessing the prognosis. The Ishikawa classification [10] is more suitable for this as it takes complications already present at the time of diagnosis into consideration (Tab. 4). There is a correlation between classification and patient prognosis: the greater the number of fulfilled minor criteria, the more severe and complicated the disease course [11, 23].

**Tab. 4:** Diagnostic criteria for Takayasu arteritis according to Ishikawa whereby the presence of two major criteria or one major and two minor criteria is highly suggestive for Takayasu arteritis

Major criteria	Symptoms consistent with Takayashu arteritis
	Lesion in the mid-portion of the left subclavian artery
	Lesion in the mid-portion of the right subclavian artery
Minor criteria	Erythrocyte sedimentation rate ≥ 20 mm/h
	Carotidynia
	Hypertension
	Aortic valve insufficiency
	Lesion in the pulmonary artery
	Lesion in the mid-portion of the common carotid artery
	Lesion in the region of the distal brachiocephalic trunk
	Lesion in the region of the descending thoracic aorta
	Lesion in the region of the abdominal arteries
	Lesion in the region of the coronary arteries

### Giant cell arteritis (synonyms: Horton’s disease and temporal arteritis)

**Fig. 6:**
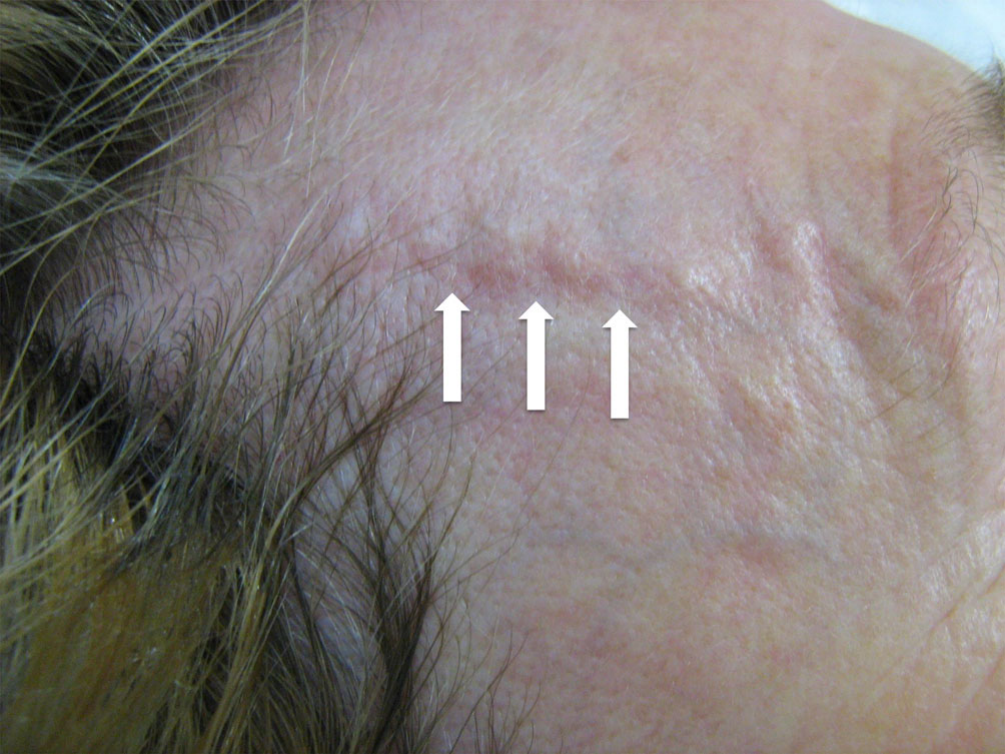
Giant cell arteritis with the classical finding of painful segmental thickening of the temporal artery (courtesy of Dr. G. Herzog)

First described in 1937 by Horton et al. giant cell arteritis is the most common form of non-infectious arteritis [cited in 10]. The aorta itself is affected by inflammatory changes in only a subgroup of patients [3]. Stenotic as well as aneurysmal lesions of the supra-aortic branches are most commonly seen. This disease predominantly affects females aged over 50 years and the prevalence increases with age. The incidence in Europe is put at 15–25 per 100,000 inhabitants per year, making it considerably higher compared to Takayasu arteritis [5]. Patients generally seek medical advice for headaches and impaired vision caused by arteritic optic nerve neuropathy. A history of jaw claudication indicating involvement of the external carotid artery and its branches is pathognomic for this disease. Fever, muscle pain, weight loss, cranial nerve deficits and double vision are rarer manifestations [7, 12]. Temporal swelling along the course of the temporal artery with tenderness on palpation is clinically striking (Fig. 6). The erythrocyte sedimentation rate (ESR) and C‑reactive protein (CRP) level are almost always both elevated. Normocytic normochromic anemia and thrombocytosis are also not uncommon. Involvement of the subclavian artery can cause arm claudication, pain at rest and ultimately lead to gangrene of the fingers and hands.

**Fig. 7:**
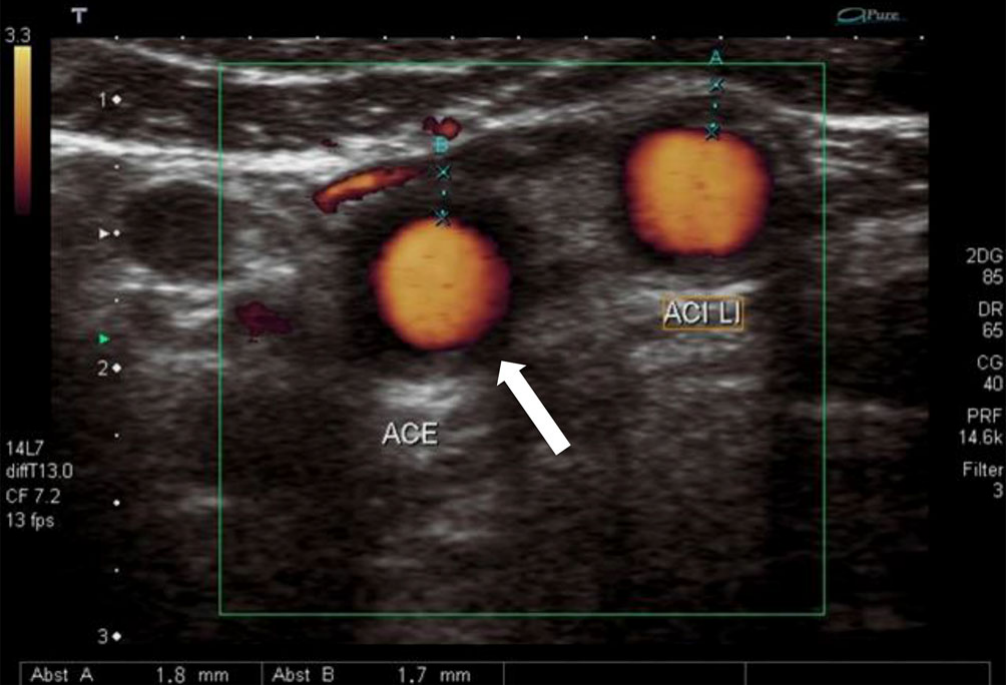
Giant cell arteritis with involvement of the extracranial carotid artery and typical halo (*arrow*) on color duplex sonography (courtesy of Dr. G. Herzog)

Early diagnosis is particularly important in view of the prognosis of ophthalmological complications [19]. As with Takayasu arteritis, the ACR has defined specific diagnostic criteria (Tab. 5). A temporal artery biopsy should be performed (prior to initiating therapy) in addition to the clinical and laboratory investigations mentioned previously. A minimum biopsy length of at least 1.5–2 cm is considered necessary due to segmental involvement of the arterial wall. Doppler and duplex ultrasound show characteristic findings (hypoechoic border or “halo”; Fig. 7, [1]); however, depending on the initial ACR score (Tab. 5) a number of studies have shown sensitivity and specificity to be significantly lower compared with temporal artery biopsy (69 and 82 vs. 93 and 91.2 %, respectively [2, 13, 24]). Having said that, a high ACR score and positive bilateral ultrasound findings generally make temporal artery biopsies unnecessary. Cross-sectional imaging methods, such as CT and MRI are well-suited to assessing vessels that cannot be visualized by ultrasound. In the case of low ACR scores and equivocal ESR, CRP and biopsy findings, PET-CT can be used to establish the diagnosis [14].

**Tab. 5:** American College of Rheumatology (ACR) criteria for giant cell arteritis and three out of five criteria need to be fulfilled for diagnosis

Age at initial manifestation > 50 years
New onset of headache
Temporal artery anomaly
Elevated erythrocyte sedimentation rate
Pathological temporal artery biopsy

### Other underlying rheumatic diseases

Aortic involvement has been described in a number of systemic rheumatic diseases; Tab. 1 shows the most important of these [2]. Suspicion of inflammatory aortic lesions should be aroused if patients with signs of an underlying rheumatic disease exhibit clinical indications of acute aortic valve insufficiency, renal or mesenteric artery occlusion, decreased perfusion to the extremities or aortic aneurysms in the absence of typical risk factors for arteriosclerosis. In some cases, however, the manifestation of typical clinical symptoms may point the way to the appropriate diagnosis (e. g. oral or genital ulcerations in AAA are specific for Behçet’s disease). Rational basic laboratory diagnostics can be helpful here from a number of perspectives: etiological classification of symptoms, type and activity of the disease processes and an evaluation of function of and damage to affected organ systems (Tab. 6).

**Tab. 6:** Rational laboratory diagnostics in the case of suspected aortitis

Basic laboratory tests	Complete blood count (including differential blood count), uric acid, creatine kinase, alkaline phosphatase, creatinine, gamma-glutamyl transferase, aspartate aminotransferase, total protein, protein electrophoresis, coagulation
	Erythrocyte sedimentation rate, C‑reactive protein
	Rheumatoid factor
	Antinuclear antibodies (ANA)
	Iron, ferritin
Special laboratory tests	Antineutrophil cytoplasmic antibodies (ANCA)
	ANA differentiation
	Double-stranded DNA antibodies (dsDNA-Ab)
	Complement, HLA typing

### Treatment

Corticosteroids form the central pillar of treatment in management of non-infectious aortitis; for example, 40–60 mg prednisone for 4 weeks followed by a gradual dose reduction is the standard treatment [18]. In addition to monitoring clinical symptoms the ESR is a good follow-up parameter [18]. Some authors recommend initial treatment with 1,000 mg methyl prednisone daily for 3 days in giant cell arteritis patients with ocular symptoms (see Ness et al. [20] for a detailed schedule). Efficacy varies from study to study and appears to depend on the extent of inflammatory lesions at the time of treatment initiation. It is fair to assume that only one in every two patients will fully respond to steroid treatment; furthermore, steroid-induced adverse effects are seen in up to 65 % of patients, causing not inconsiderable comorbidities. In such situations the use of other steroid-sparing substances can be considered. Three randomized placebo-controlled studies on the use of methotrexate in giant cell arteritis yielded conflicting results, while one meta-analysis found a certain advantage conferred by 10–15 mg/week in terms of reducing recurrence rates and steroid dosage [15]. A randomized trial that combined steroids with infliximab also revealed no advantages compared with placebo [7]. A reduction in steroid maintenance dose in patients with Takayasu arteritis was possible with both azathioprine (2 mg/kg body weight/day) and methotrexate (20–25 mg/week [8, 31]). To date, cyclophosphamide has been tested only in a small study on adults with steroid-refractory Takayasu arteritis. The administration of low-dose acetylsalicylic acid (75–150 mg/day) reduced the manifestation of cardiovascular and cerebrovascular events in giant cell arteritis patients [14, 19].

Interventional and conventional surgical management of arterial occlusion or aneurysms are guided by clinical symptoms and the basic principles of arteriosclerosis treatment. Although the results of reconstructive interventions are good, the need for surgical revision is significantly increased in the long term [4]. Several authors recommend performing revascularization when inflammatory activity is at its lowest [4, 16]. The restenosis rate following interventional procedures, such as angioplasty or angioplasty and stents is higher compared with surgical procedures [4, 12, 17, 23]. Data on the use of drug-eluting stents or drug-eluting balloons are lacking. Due to the rareness of the disease there is a simple lack of evidence to make specific recommendations on the selection of surgical or endovascular procedures, the type of stents or stent grafts to be used or resection in the case of aneurysms.

### Chronic idiopathic periaortitis

Since this disease was first described evidence has grown to suggest that inflammatory abdominal aortic aneurysms (IAAA) are not, as originally assumed, a distinct entity but belong to a group consisting of three symptom complexes with the same etiology: (1) inflammatory AAA, (2) idiopathic retroperitoneal fibrosis (Ormond’s disease) and (3) a combination of these two [6, 25].

#### Inflammatory aortic aneurysm

The term inflammatory aneurysm was first used by Walker in 1972 [cited in 27] who described a group of aortic aneurysms intraoperatively characterized by a thickened wall, marked perianeurysmal and retroperitoneal fibrosis and adhesions to adjacent organs. Inflammatory aneurysms account for approximately 3–11 % of all AAAs and are predominantly seen in males. The average age of onset is striking in that it is 5–10 years lower that in patients with non-inflammatory aneurysms [25]. Likewise, the proportion of smokers in this group was significantly higher at 77–100 % [21]. There is evidence that a familial tendency to aneurysm formation is found 10 times more frequently in patients with inflammatory aneurysm, leading to the assumption of a genetic predisposition [18].

Our understanding of the processes that underlie aneurysm formation has significantly increased in recent years. It is becoming apparent that a combination of genetically predisposing factors and certain pathophysiological factors cause aneurysm genesis through inflammatory degradation of the aortic wall. Thus, according to current knowledge, it is likely that inflammatory AAA does not represent a separate disease entity but is a particularly extreme variant of this inflammatory process [21]. Although the etiology of the clearly antigen-triggered inflammatory response has not been fully elucidated as yet, possibilities include degradation products from lipids deposited in the wall, elastin degradation products or infectious triggers (e.g. *Chlamydia pneumoniae*, herpes simplex virus and cytomegalovirus) [11, 22, 27, 28]. These lead in the further course to the disintegration of the extracellular matrix via the release and activation of proteolytic enzymes from immune cells, e. g. metallomatrix proteases. Classically, bacterial pathogens are not detected on histopathological and microbiological analysis of the aneurysm wall [3]. The onset of inflammatory aneurysms is virtually always associated clinically with abdominal, back or flank pain (65–90 %), which are seen significantly less frequently in non-inflammatory aneurysms (8–18 % [29]). Lack of appetite and weight loss are seen in almost 50 % of patients. In addition, the ESR is generally strikingly elevated (40–88 %). Although leukocytosis and fever are seen in isolated cases they do not represent typical elements of the clinical picture [25]. The occurrence of comorbidities, such as arteriosclerosis, coronary heart disease, hypertension and diabetes is the same as in patients with non-inflammatory aneurysms; however, ureteral involvement in periaortic inflammation, which is seen in up to 50 % of patients and not infrequently causes chronic obstructive postrenal renal dysfunction (18–21 %), is clinically indicative.

Inflammatory AAA is diagnosed intraoperatively in the majority of cases, although both CT [9] and ultrasound (halo) show good diagnostic accuracy [28]. According to Sterpetti et al. ureteral involvement is best assessed using a combination of excretory pyelography and CT [25]. The inflammatory periaortic mass can also be well visualized using contrast-enhanced MRI, whereby this method yields other data necessary for perioperative planning to a lesser extent [30]. The inflammatory etiology of inflammatory aortic aneurysms makes primarily drug-based therapy with corticosteroids appear reasonable. Indeed, there are individual reports on the complete resolution of clinical symptoms and all inflammatory changes to the aortic wall and retroperitoneum under steroid therapy [29]; however, a reduction in aneurysm diameter has not been observed. If the role of arteriosclerotic changes in the formation of inflammatory aneurysm are taken into consideration, the resulting reduction in risk factors assumes crucial importance. Abstention from tobacco use in particular appears to have a positive effect on disease course [28]. The indications for surgical treatment are assessed in much the same way as in non-inflammatory aneurysms, whereby the frequent onset of pain tends to prompt early surgery.

The strategy of open aortic repair differs from non-inflammatory aneurysms in that dissection should be kept to a minimum. Attempts to detach inflammatory adhesions involving adjacent organs, such as the duodenum, sigmoid colon, vena cava or ureters from the aneurysm can cause damage to these organs and initially contributed to significantly higher morbidity and mortality compared with non-inflammatory aneurysms [1, 21]. As early as in 1985, Crawford et al. recommended infradiaphragmatic cross-clamping to create a proximal anastomosis [1]. The inflamed wall can easily tear and should be reinforced with polytetrafluoroethylene (PTFE) pledgets. Thus, improvements in surgical techniques have led to a gradual reduction in the mortality rate, which was virtually comparable with non-inflammatory AAA in the 1990s (6.8–11 %; [26, 28, 32]). Anatomy permitting, there appear to be no other obstacles to endovascular treatment. Initial reports show good results in terms of mortality and morbidity, comparable with those achieved with endovascular treatment of arteriosclerotic aneurysms [13, 28]; however, it remains unclear why a regression in fibrotic periaortitis is seen significantly less frequently following endovascular treatment [26]. It is possible that the endografts themselves are capable of triggering an inflammatory reaction in the aortic wall and directly adjacent structures, as observed following treatment of non-inflammatory aneurysms. Thus, the indications for *E*ndo*V*ascular *A*neurysm *R*epair (*EVAR*), at least in IAAA with ureteral involvement, should be considered from a critical perspective [15]. A systematic review of the Cochrane working group on this topic is underway.

#### Ormond’s disease (retroperitoneal fibrosis)

This disease is characterized by the same clinical, radiological and etiological features as IAAA, differing only in that there is no aneurysm formation [6]. The overlap and possible combinations in the severity of individual symptoms are by nature fluid. Obstructive ureteral complications, which can usually be managed by placement of a splinting catheter, take the foreground. Otherwise, treatment is limited to the use of corticosteroids in combination with immunosuppressants, such as azathioprine, cyclophosphamide and methotrexate, although it is difficult to predict individual responses to these drugs [29].

## Conclusion

Non-infectious inflammatory processes of the aorta and its branches are associated with severe sequelae. Initially, diagnosis may be challenging due to the nonspecific clinical presentation. Thus, particularly in surgical disciplines it is of crucial importance to “think of it in the first place.” Prompt intensive drug treatment can reduce long-term morbidity in many cases. Vascular surgery plays a crucial role within the interdisciplinary treatment team.
